# Association of salivary matrix metalloproteinase-8 with metabolic syndrome in women at high GDM risk

**DOI:** 10.1186/s12903-026-09564-0

**Published:** 2026-08-13

**Authors:** Hanna Poulsen, Jukka H. Meurman, Hannu Kautiainen, Emilia Huvinen, Saila Koivusalo, Taina Tervahartiala, Ella Brandt, Timo Sorsa, Johan G. Eriksson

**Affiliations:** 1 Department of Oral and Maxillofacial Diseases, University of Helsinki, Biomedicum Helsinki, 1 Haartmaninkatu 8, P.O. Box 63, Helsinki, FI-00014 Finland; 2 Department of General Practice and Primary Health Care, University of Helsinki and Helsinki University Hospital, Helsinki, Finland; 3 Department of Obstetrics and Gynaecology, University of Helsinki, Helsinki, Finland; 4 Department of Obstetrics and Gynaecology, Helsinki University Hospital, Helsinki, Finland; 5 Division of Periodontology, Department of Dental Medicine, Karolinska Institutet, Huddinge, Sweden; 6 Department of General Practice and Primary Health Care, University of Helsinki, Helsinki, Finland; 7 Folkhälsan Research Center, Helsinki, Finland

**Keywords:** Gestational diabetes, Insulin resistance, Beta cell function, Observational study

## Abstract

**Background:**

To assess associations between metabolic syndrome (MetS), insulin resistance, and periodontal diseases with salivary active matrix metalloproteinase-8 (aMMP-8) in metabolically high-risk women.

**Methods:**

A total of 112 women with a history of high gestational diabetes mellitus (GDM) risk were examined 4–6 years postpartum. MetS was defined using National Cholesterol Education Program Adult Treatment Panel III criteria, and insulin resistance was estimated using HOMA-IR. Oral health was assessed through clinical and radiographic examination, registering dental plaque, bleeding on probing, attachment loss, and number of teeth. Salivary levels of active and total matrix metalloproteinase-8 (tMMP-8) were determined using immunofluorescence assay and ELISA, respectively. Tissue inhibitor of metalloproteinase-1 (TIMP-1) levels were also measured. Questionnaires provided information on participants’ oral health habits and symptoms.

**Results:**

Periodontitis and a high periodontal inflammatory burden index correlated with the tMMP-8/TIMP-1 ratio. The aMMP-8 levels were associated with visible plaque index, HOMA-IR and MetS. The aMMP-8/TIMP-1 ratio correlated with the visible plaque index and it showed a non-significant trend toward higher levels with MetS. The tMMP-8 were associated with HOMA-IR and MetS.

**Conclusions:**

Elevated tMMP-8 concentrations were associated with periodontal diseases among high-risk women, further supporting the potential role of salivary aMMP-8 as a biomarker of insulin resistance and MetS in this population.

## Introduction

Gestational diabetes mellitus (GDM) is a well-known indicator of a woman’s future cardiometabolic. GDM is associated with a 10-fold increased risk of type 2 diabetes (T2D) later in life, as well as increased risk of metabolic syndrome (MetS) [1]. Central obesity, hyperglycaemia, elevated blood pressure, and hyperlipidaemia are characteristic features of MetS, and its prevalence is rising globally [2]. MetS also increases the risk for T2D and cardiovascular diseases (CVD) [2, 3]. Obesity and insulin resistance are the main underlying risk factors for MetS however, GDM is also a well-known risk factor for MetS [2, 4, 5]. Periodontal disease has been associated with MetS, obesity, T2D and GDM [6–8]. Furthermore, all these conditions share a state of low-grade inflammation [9–11].

Matrix metalloproteinase-8 (MMP-8) is a collagenolytic enzyme, also known as collagenase-2, which is released by selective degranulation especially from neutrophils. It plays an essential role in normal tissue remodelling, and in several inflammatory processes. In addition to MMP-8, several other MMPs and their inhibitors, tissue inhibitors of metalloproteinases (TIMPs), and numerous other extracellular matrix-modifying mediators participate in these events. TIMPs are crucial in regulating the degradation of extracellular matrix components, and thus a balanced molar ratio between MMPs and TIMPs is considered an important indicator of healthy tissue [12]. In inflammatory cascades, MMP-8 levels increase and the balance between MMPs and TIMPs is disturbed. In tissues, MMP-8 exists in two forms, active collagenolytic MMP-8 (aMMP-8) and inactive/latent non-collagenolytic pro-MMP-8 (proMMP-8). Together, these forms constitute total MMP-8 (tMMP-8). The collagenolytic tissue destruction observed in conditions such as periodontitis is principally mediated by aMMP-8 [13, 14].

Several studies have described an association between periodontitis and elevated aMMP-8 levels [15–18]. In people with diabetes MMP-8 levels are elevated in gingival crevicular fluid (GCF) and saliva [19, 20], especially those with periodontitis and poorly controlled T2D [21]. Recent findings from an adult population have linked elevated aMMP-8 concentrations with MetS [22]. Elevated MMP-8 levels are associated with both periodontitis and GDM, suggesting a potential link between oral health and the development of GDM [23]. Evidence suggests that aMMP-8 may reflect periodontal inflammation more specifically than tMMP-8 [14, 24]. Indeed, elevated aMMP-8 levels could have the potential utility in detecting prediabetes and diabetes among people with periodontal disease, those who are overweight, and those with increasing age [21, 22, 25]. Additionally, it could have potential utility as a grading and staging biomarker in the new periodontitis classification [21, 25–27].

Women at increased risk of GDM differ from other high cardiometabolic risk groups by usually being younger and exhibiting an early-onset risk detected during the metabolic stress of pregnancy. Therefore, little is known about MMP-8 in this specific group. Furthermore, cardiometabolic health has been less studied in women than in men. This study explored whether there were potential associations between salivary MMP-8, both aMMP-8 and tMMP-8 levels, respectively, with metabolic and periodontal status among metabolically high-risk women with a history of GDM or a high risk of GDM. Specifically, we investigated whether salivary aMMP-8 could serve as a biomarker for insulin resistance, MetS, and periodontitis among these metabolically high-risk women. We hypothesized that women with MetS, insulin resistance, and periodontal diseases have higher salivary aMMP-8 levels than women without these conditions.

## Materials and methods

**Fig. 1:**
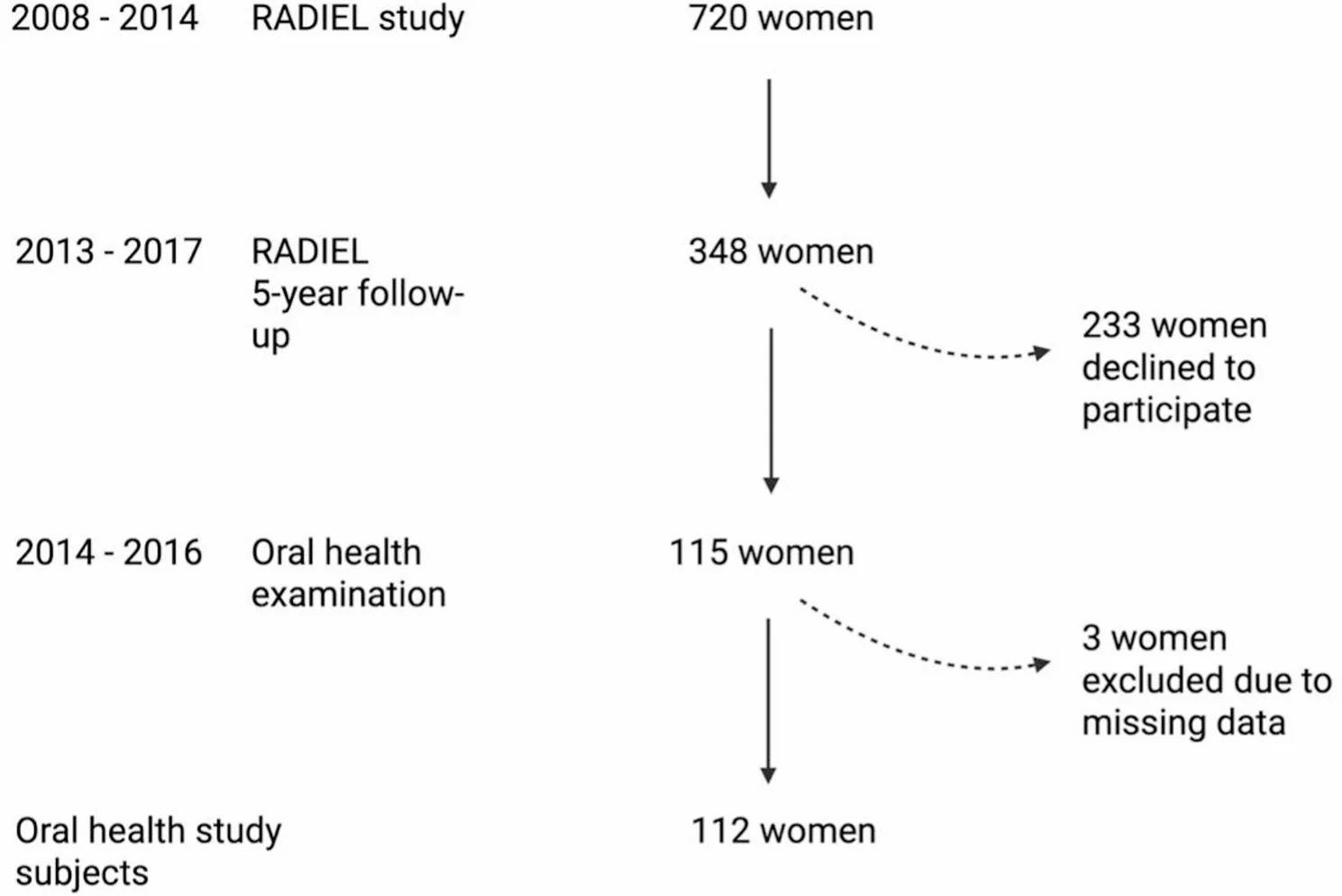
Study population. Created by Biorender.com

This study is a cross-sectional substudy nested within the prospective cohort of the Finnish Gestational Diabetes Prevention Study (RADIEL). The publication by Rönö, Stach-Lempinen [28] provides more detailed information of the study design. In brief, the RADIEL study was a randomized controlled multi-centre intervention trial involving 720 women, who were recruited between 2008 and 2014. The original study aimed to prevent GDM through lifestyle intervention, including physical activity and dietary modifications. The women were enrolled before reaching their 20th week of pregnancy, all being at high risk for GDM due to either obesity (BMI ≥ 30 kg/m²) or a prior history of GDM. In this follow-up study, participants were contacted 4 to 6 years after childbirth and invited to participate in the follow-up study. Altogether 348 women responded, and they were further invited to an oral health investigation. 115 women took part in the oral health examination but three of them were excluded due to missing data. Therefore, the current cross-sectional oral health study involved 112 non-pregnant women, aged 27 to 49 years, from the original RADIEL study. Figure 1 presents the study flow chart (Fig. 1).

During the follow-up visit, study nurses obtained anthropometric measurements (weight, height, abdominal waist circumference) and collected venous blood samples. Biochemical analyses by the hospital laboratory provided data on glucose metabolism (glycated haemoglobin (HbA1c), fasting glucose, fasting insulin), lipid metabolism (low-density lipoprotein (LDL), high-density lipoprotein (HDL), total cholesterol, triglycerides (TG)), and inflammatory markers (high-sensitivity C-reactive protein). Blood pressure was measured in the right arm in the sitting position with a sphygmomanometer. The multi-frequency bio-impedance measurement (InBody 3.0, Biospace Co., Ltd., Seoul, Korea) provided data on body composition [29].

Participants fasted overnight before the venous blood samples were drawn. Plasma glucose levels were measured using an enzymatic hexokinase assay (Roche Diagnostics, Basel, Switzerland). LDL, HDL, TG, and total cholesterol were analysed through standard hospital laboratory methods. HbA_1c_ was measured with an immunoturbidimetric analyser. A 2-hour 75 g oral glucose tolerance test provided the data for glucose tolerance. Insulin resistance was determined by calculating the Homeostatic Model Assessment for Insulin Resistance (HOMA-IR) index using fasting glucose and insulin concentrations, as described by Matthews et al. [30].

MetS was defined according to the National Cholesterol Education Program Adult Treatment Panel III (NCEP/ATP III) criteria. The diagnosis required fulfilling at least three of the following five criteria: fasting plasma glucose ≥ 5.6 mmol/L, waist circumference > 88 cm, systolic blood pressure ≥ 130 mmHg or diastolic blood pressure ≥ 85 mmHg, triglycerides ≥ 1.7 mmol/L, and HDL cholesterol < 1.29 mmol/L [2]. 

A complete oral health examination and panoramic radiographs of the jaws provided data on dental and periodontal health. Before the dental examination, stimulated saliva samples were collected by paraffin wax stimulation and stored at -75 °C until further analysis [31]. A single examiner (HP) conducted the examinations, uninformed of the participants’ metabolic parameters. Data on number of teeth, dental, periodontal, and mucosal health were registered. Periodontal examinations included Bleeding on Periodontal Probing (BOP) index and the Visible Plaque Index (VPI), calculated according to Ainamo and Bay [32], as well as measurements of probing pocket depth, gingival recession, and clinical attachment loss (CAL) measurements performed at six sites per tooth using a WHO probe. Periodontitis was classified according to the updated case definitions proposed by Eke et al. as mild, moderate, or severe [33]. Total Dental Index (TDI), Decayed, Missing, and Filled Teeth (DMFT) index, and Periodontal Inflammatory Burden Index (PIBI) according to Lindy et al., were calculated [34]. The panoramic X-rays were analysed for signs of infections of the teeth and marginal alveolar bone loss. A structured questionnaire collected data on the women’s oral health habits, potential symptoms, and the perception of their oral health. After the examination, all subjects received information on their oral health and jaw radiographic findings. Those women in need of treatment were advised to make a dental appointment.

Salivary tMMP-8 and TIMP-1 concentrations were determined using commercial ELISA kits according to the manufacturer’s instructions and previously published protocols [35]. The aMMP-8 levels from saliva samples were measured using a time-resolved immunofluorescence assay (IFMA) as previously described by Keskin, Rintamarttunen [36]. The assays were performed using established and standardized protocols routinely used in the laboratory. The detection limit for the aMMP-8 IFMA was 0.08 ng/mL, and an inter-assay coefficient of variation of 7.3% has been reported for the assay. The detection limit for the tMMP-8 ELISA was 0.013 ng/mL. All samples were successfully analysed using both assays, and no samples had values below the respective detection limits. Intra-assay coefficients of variation were not determined in the present study, as replicate measurements within the same assay run were not performed. Similarly, the inter-assay coefficient of variation for the tMMP-8 ELISA was not determined in the present study, as the samples were not analysed in multiple independent assay runs.

### The molar ratios

For the calculation of aMMP-8 IFMA/TIMP-1 or tMMP-8 ELISA/TIMP-1 molar ratios, the levels were converted to mol per litre and based on the ability of MMPs to form complexes with TIMPs at a 1:1 stoichiometric ratio [12].

### Statistical analyses

Summary statistics are described using mean and standard deviation (SD), or median and interquartile range (IQR). Age-, education-, and smoking-adjusted partial Pearson correlation coefficients were calculated, and Sidak-adjusted p-values were used to account for multiple comparisons. Comparisons between participants with and without metabolic syndrome were performed using the Mann–Whitney U test. All analyses were performed using STATA software, version 18 (StataCorp LP, College Station, TX). Variables with skewed distributions were log-transformed when necessary.

## Results

The basic characteristics of the study participants are presented in Table 1. The study included 112 women. The mean age was 39.3 years, and MetS was diagnosed in 20.5% of the participants (*n* = 23). Radiographic examinations revealed no clinically significant infectious findings. Only five of the 112 women (4.5%) showed radiographic findings suggestive of mild inflammation.

**Table 1:** Basic characteristics of the subjects at 5-year follow-up

Variable	ALL *N* = 112	Non-MetS *N* = 89	MetS *N* = 23
Age (years), mean (SD)	39.3 (5.1)	39.1 (5.1)	40.3 (5.2)
Weight (kg), mean (SD)	86.8 (19.9)	82.5 (18.6)	103.1 (16.3)
BMI (kg/m^2^), mean (SD)	31.4 (6.8)	30.0 (6.6)	36.7 (4.6)
Waist (cm), mean (SD)	104 (17)	100 (16)	117 (12)
Body fat %, mean (SD)	38.1 (9.5)	36.4 (9.7)	44.6 (5.3)
Education years, mean (SD)	14.9 (1.9)	15.0 (1.8)	14.5 (2.0)
Smoking, n (%)	13 (12)	13 (15)	0 (0)
Physical activity, (min/week), median (IQR)	60 (30–150)	90 (40–180)	60 (30–90)
HbA1c, (%), mean (SD)	5.51 (0.30)	5.47 (0.30)	5.67 (0.27)
Fasting plasma glucose (mmol/L), mean (SD)	5.15 (0.55)	5.04 (0.50)	5.54 (0.57)
Insulin (mU/L)	10.22 (6.53)	8.92 (5.50)	15.23 (7.80)
HOMA-IR	2.39 (1.65)	2.04 (1.38)	3.72 (1.95)
Total cholesterol (mmol/L), mean (SD)	4.59 (0.74)	4.63 (0.73)	4.44 (0.75)
HDL cholesterol (mmol/L), mean (SD)	1.54 (0.38)	1.63 (0.35)	1.21 (0.29)
LDL cholesterol (mmol/L), mean (SD)	2.91 (0.70)	2.90 (0.71)	2.97 (0.65)
Total triglycerides (mmol/L), mean (SD)	0.89 (0.38)	0.82 (0.33)	1.17 (0.44)
High-sensitivity CRP (mg/L), mean (SD)	2.54 (4.03)	1.96 (2.51)	4.79 (7.07)
Impaired glucose regulation, n (%)			
Impaired fasting glucose (IFG), n (%)	5 (4)	4 (4)	1 (4)
Impaired glucose tolerance (IGT), n (%)	7 (6)	4 (4)	3 (13)
Diabetes mellitus (DM), n (%)	4 (4)	1 (1)	3 (13)
Blood pressure, (mmHg)			
Systolic	125 (13)	118 (12)	128 (13)
Diastolic	79 (9)	76 (8)	89 (7)

*SD* standard of deviation, *BMI* body mass index, weight in kg / height in meters^2^, *IQR* interquartile range, *HbA1c* glycated hemoglobin, *HOMA-IR* homeostasis model assessment of insulin resistance, *HDL* high density lipoprotein, *LDL* low density lipoprotein, *CRP* C-reactive protein

Table 2 presents the findings of the partial correlation analyses between systemic parameters and aMMP-8 and tMMP-8 levels and ratios. Insulin concentrations and HOMA-IR values were positively correlated with salivary aMMP-8 levels/concentrations (*r* = 0.34, *p* < 0.01; *r* = 0.35, *p* < 0.01, respectively) as well as salivary aMMP-8/TIMP-1 molar ratio (*r* = 0.28, *p* < 0.05). Body fat percentage correlated with tMMP-8 (*r* = 0.28, *p* < 0.05). Women with MetS showed higher unadjusted aMMP-8 levels (*p* = 0.035), and trends toward higher aMMP-8/TIMP-1 molar ratio, and tMMP-8 levels (*p* = 0.066) (Table 3).

**Table 2:** Results from partial correlation analyses of systemic parameters with respect to the matrix metalloproteinase levels and molar ratios, adjusted with age, education, and smoking status

	aMMP-8 *r*(95% CI)	TIMP-1 *r*(95% CI)	aMMP-8/ TIMP-1 *r*(95% CI)	tMMP-8 *r*(95% CI)	tMMP-8/TIMP-1 *r*(95% CI)
HbA1c, (%)	-0.06 (-0.24 to 0.13)	0.03 (-0.16 to 0.21)	-0.08 (-0.26 to 0.11)	0.14 (-0.04 to 0.32)	0.10 (-0.09 to 0.28)
Fasting glucose	0.16 (-0.03 to 0.34)	0.01 (-0.17 to 0.20)	0.13 (-0.06 to 0.30)	-0.01 (-0.20 to 0.17)	-0.02 (-0.20 to 0.17)
Insulin (mU/L)	0.34 (0.17 to 0.50)**	0.05 (-0.14 to 0.23)	0.27 (0.09 to 0.44)	0.15 (-0.04 to 0.33)	0.12 (-0.07 to 0.30)
HOMA-IR	0.35 (0.18 to 0.50)**	0.05 (-0.13 to 0.24)	0.28 (0.10 to 0.44)*	0.14 (-0.04 to 0.32)	0.11 (-0.08 to 0.29)
Total cholesterol	-0.18 (-0.35 to 0.00)	0.15 (-0.04 to 0.33)	-0.22 (-0.39 to -0.03)	-0.15 (-0.33 to 0.04)	-0.18 (-0.36 to 0.00)
HDL cholesterol	-0.20 (-0.37 to -0.02)	-0.02 (-0.21 to 0.16)	-0.16 (-0.34 to 0.03)	-0.18 (-0.35 to 0.01)	-0.16 (-0.33 to 0.03)
LDL cholesterol	-0.06 (-0.25 to 0.12)	0.12 (-0.07 to 0.30)	-0.11 (-0.29 to 0.08)	-0.12 (-0.30 to 0.07)	-0.14 (-0.32 to 0.05)
Total triglycerides	0.21 (0.03 to 0.38)	0.07 (-0.12 to 0.25)	0.15 (-0.03 to 0.33)	0.18 (-0.01 to 0.35)	0.14 (-0.05 to 0.31)
CRP	0.10 (-0.09 to 0.28)	0.00 (-0.18 to 0.19)	0.07 (-0.12 to 0.25)	0.14 (-0.05 to 0.32)	0.11 (-0.08 to 0.29)
BMI	0.23 (0.05 to 0.40)	0.07 (-0.12 to 0.25)	0.16 (-0.02 to 0.34)	0.24 (0.06 to 0.41)	0.18 (-0.00 to 0.36)
Waist	0.23 (0.05 to 0.40)	0.15 (-0.04 to 0.32)	0.13 (-0.06 to 0.31)	0.23 (0.05 to 0.40)	0.15 (-0.04 to 0.33)
Body fat %,	0.25 (0.07 to 0.42)	0.04 (-0.14 to 0.23)	0.19 (0.00 to 0.36)	0.28 (0.10 to 0.44)*	0.22 (0.03 to 0.39)
Systolic	0.09 (-0.10 to 0.27)	0.11 (-0.08 to 0.29)	0.02 (-0.16 to 0.21)	0.24 (0.05 to 0.40)	0.16 (-0.03 to 0.33)
Diastolic	0.12 (-0.07 to 0.30)	0.11 (-0.07 to 0.29)	0.04 (-0.14 to 0.23)	0.22 (0.04 to 0.39)	0.15 (-0.03 to 0.33)

*HbA1C* glycated haemoglobin, *HOMA-IR* homeostasis model assessment of insulin resistance, *HDL* high density lipoprotein, *LDL* low-density lipoprotein, *CRP* high-sensitivity C-reactive protein, *BMI* body mass index, *aMMP-8* active-matrix metalloproteinase-8, *tMMP-8* total-matrix metalloproteinase-8, *TIMP-1* tissue-inhibitor matrix metalloproteinase-1

Sidak adjusted *p < 0.05, **p < 0.01, ***p < 0.001

**Table 3:** Levels of salivary aMMP-8, tMMP-8 and TIMP-1 and their ratio of the women (*N* = 112) without and with metabolic syndrome (MetS)

	Non-MetS *n* = 89 Median (IQR)	MetS *n* = 23 Median (IQR)	*p*-value
aMMP-8 (ng/mL)	100 (41, 272)	188 (68, 654)	0.035
TIMP-1 (ng/mL)	98 (71,132)	100 (75, 130)	0.71
aMMP-8/ TIMP-1	0.53 (0.19, 1.50)	0.77 (0.30, 2.69)	0.072
tMMP-8 (ng/mL)	75 (41, 195)	123 (94, 262)	0.066
tMMP-8/TIMP-1	0.37 (0.15, 1.18)	0.65 (0.31, 1.10)	0.12

*IQR* interquartile range, *aMMP-8* active-matrix metalloproteinase-8, *tMMP-8* total-matrix metalloproteinase-8, *TIMP-1* tissue-inhibitor matrix metalloproteinase-1

Partial correlation analyses of oral health parameters and the MMPs revealed significant associations between oral health parameters and aMMP-8, aMMP-8/TIMP-1, tMMP-8, and tMMP-8/TIMP-1 as shown in Table 4. The strongest associations were observed between PIBI values and periodontitis with tMMP-8/TIMP-1-molar ratio (*r* = 0.37 and *r* = 0.35, respectively, *p* < 0.001) and between PIBI and tMMP-8 (*r* = 0.36, *p* < 0.001). In addition, VPI was associated with aMMP-8 (*r* = 0.26, *p <* 0.05), aMMP-8/TIMP-1 molar ratio (*r* = 0.33, *p* < 0.01) and TIMP-1 (*r*=-0.29, *p* < 0.05). BOP-index was associated with tMMP-8 (*r* = 0.28, *p* < 0.05) and the tMMP-8/TIMP-1 molar ratio (*r* = 0.25, *p* < 0.05), while TDI was associated with the tMMP-8/TIMP-1 molar ratio (*r* = 0.27, *p* < 0.05).

**Table 4:** Results from partial correlation analyses of oral health parameters with respect to the matrix metalloproteinase levels and molar ratios, adjusted with age, education, and smoking status

	aMMP-8 *r* (95% CI)	TIMP-1 *r* (95% CI)	aMMP-8/ TIMP-1 *r* (95% CI)	tMMP-8 *r* (95% CI)	tMMP-8/TIMP-1 *r* (95% CI)
TDI	0.10 (-0.08 to 0.28)	-0.16 (-0.34 to 0.03)	0.14 (-0.05 to 0.32)	0.23 (0.05 to 0.40)	0.27 (0.09 to 0.43)*
DMFT	0.02 (-0.17 to 0.20)	-0.09 (-0.27 to 0.10)	0.03 (-0.16 to 0.21)	0.18 (-0.00 to 0.36)	0.17 (-0.02 to 0.34)
PIBI	0.15 (-0.03 to 0.33)	-0.10 (-0.28 to 0.09)	0.17 (-0.02 to 0.35)	0.36 (0.19 to 0.51)***	0.37 (0.20 to 0.52)***
BOP-Index	-0.10 (-0.28 to 0.08)	0.07 (-0.12 to 0.25)	-0.11 (-0.29 to 0.08)	0.28 (0.10 to 0.45)*	0.25 (0.07 to 0.42)*
VPI	0.26 (0.08 to 0.42)*	-0.29 (-0.45 to -0.11)*	0.33 (0.15 to 0.49)**	0.18 (-0.00 to 0.36)	0.27 (0.09 to 0.44)*
Periodontitis	0.14 (-0.04 to 0.32)	-0.07 (-0.25 to 0.12)	0.15 (-0.04 to 0.33)	0.34 (0.17 to 0.50)**	0.35 (0.18 to 0.51)***

*TDI* total dental index, *DMFT* decayed, missing, and filled teeth, *PIBI* periodontal inflammatory burden index, *BOP* bleeding on probing, *VPI* visible plaque index, *aMMP-8* active-matrix metalloproteinase-8, *tMMP-8* total- matrix metalloproteinase-8, *TIMP-1* tissue-inhibitor matrix metalloproteinase-1

Sidak adjusted *p < 0.05, **p < 0.01, ***p < 0.001

## Discussion

To the best of our knowledge, this study is the first to investigate oral health and salivary aMMP-8 and tMMP-8 levels among women at high risk of GDM during the first years after pregnancy. Thomas et al. Thomas, Joseph [22] recently demonstrated that elevated aMMP-8 levels in mouth rinse samples correlated with metabolic parameters in a metabolic adult study sample [22]. The present findings are consistent with the observations reported by Thomas et al. [22] and suggest that a similar association may also exist among women with a history of, and high risk for, GDM. All women in our study were at high cardiometabolic risk, and according to our data, salivary aMMP-8 may serve as a biomarker of insulin resistance and MetS among these high-risk women. Our study differs from that of Thomas et al. in the biological sample analysed, as we used saliva whereas Thomas et al. utilised mouth-rinse samples. Among oral-fluid biomarkers, mouthrinse aMMP-8 has been reported to be a highly accurate biomarker for periodontal disease detection [22, 26, 37, 38]. In this study, tMMP-8 and tMMP-8/TIMP-1 molar ratio correlated with the presence of periodontitis and aMMP-8/TIMP-1 molar ratio with VPI. Overall, the MMP-8/TIMP-1 molar ratio reflects the balance between MMP-8 activity and its inhibition by TIMP-1. VPI reflects dental plaque accumulation and the oral microbial burden. Oral microbes, particularly anaerobic periodontal pathogens, are capable of converting latent proMMP-8 into its active form (aMMP-8) [39, 40].

Previous studies have further proposed that elevated aMMP-8 levels may be associated with prediabetes or diabetes among overweight subjects with periodontitis and higher age [18, 41]. A cross-sectional study reported an independent and weak association with salivary aMMP-8 among subjects with MetS and periodontitis [22]. These findings are in agreement with our results. It should be noted that all participants in our study were Scandinavian women, whereas the study by Grigoriadis et al. involved Greek participants and the study by Thomas et al. included Indian men and women [18, 22, 41].

Another cross-sectional study from two longitudinal twin studies reported an association between obesity and elevated serum aMMP-8 levels [42]. These subjects were 22–36 years old twins and therefore the study population differed markedly from our population. In their prospective study Carbone et al. [43] found a correlation between circulating MMP-8 and severity of MetS among subjects with MetS. The underlying mechanism may involve aMMP-8, which can cleave insulin receptors and therefore the aMMP-8 dependent insulin-receptor processing may enhance insulin resistance [42, 44]. However direct evidence in humans is still missing.

One of the key strengths of our study was the comprehensive oral health assessment, including full-mouth panoramic X-rays. The fact that all examinations were conducted by a single specialist was an additional strength, as it minimized variability between examiners. Another asset is the detailed metabolic evaluation of the subjects. Moreover, all participants belonged to a relatively homogenous Finnish population.

However, our study has some limitations. We lacked information on the oral health status of the participants during their index pregnancy five years earlier. This is a limitation, as it restricts our ability to perform longitudinal analyses or establish causality. Additionally, we did not have annually recorded medical data, so it remains unclear when the subjects developed MetS. We also lacked information on medication use at the time of the study, which may have influence MMP-8 levels or MetS components. We recognize that there is an overlap in aMMP-8 concentrations between those with MetS and those without, which needs to be taken into account when considering aMMP-8 as a biomarker for MetS. Intra-examiner reproducibility of the periodontal measurements was not formally assessed, and therefore, the potential for measurement variability cannot be excluded. Another limitation is that we were unable to evaluate the potential for selection bias, as oral health data were not available for participants who were lost to follow-up or declined participation in the oral health study. Our recruitment criteria may restrict the generalizability of the findings, though it can also be viewed as a strength, as to our knowledge, this was the first study to specifically investigate this high-risk group of women. Nonetheless, our conclusions apply only to this particular group and cannot be generalized to other populations. Furthermore, the sample size was small because many women from the original cohort declined to take part in the oral health assessment, which may have limited the study’s power to detect modest effect sizes.

In conclusion, our findings provide some support for our hypothesis, with higher salivary aMMP-8 concentrations observed in women with MetS and insulin resistance, while some between-group comparisons did not reach statistical significance. Salivary aMMP-8 analysis could potentially be used in clinical practice to screen for an increased cardiometabolic risk although further studies are needed. Furthermore, women with periodontal diseases had higher salivary tMMP-8 levels than women without these conditions.

## Data Availability

The datasets used and/or analysed during the current study are available from the corresponding author upon reasonable request.

## Abbreviations

BMI: Body Mass Index
BOP: Bleeding on Probing
CAL: Clinical Attachment Loss
CVD: Cardiovascular Disease
CRP: C-reactive Protein
DMFT: Decayed, Missing, and Filled Teeth
ELISA: Enzyme-linked Immunosorbent Assay
GDM: Gestational Diabetes mellitus
HbA_1c_: Glycated Haemoglobin
HDL: High-Density Lipoprotein
HOMA-IR: Homeostatic Model Assessment of Insulin Resistance
IFG: Impaired Fasting Glucose
IGT: Impaired Glucose Tolerance
IFMA: Immunofluorometric Assay
IQR: Interquartile Range
LDL: Low-Density Lipoprotein
MetS: Metabolic Syndrome
MMP: Matrix Metalloproteinase
aMMP-8: Active Matrix Metalloproteinase-8
tMMP-8: Total Matrix Metalloproteinase-8
NCEP/ATP III: National Cholesterol Education Program/Adult Treatment Panel III
TIMP-1: Tissue Inhibitor Matrix Metalloproteinase-1
TG: Triglyceride
PIBI: Periodontal Inflammatory Burden Index
RADIEL: The Finnish Gestational Diabetes Prevention Study
SD: Standard Deviation
TDI: Total Dental Index
T2D: Type 2 Diabetes
VPI: Visible Plaque Index
